# Mapping species distributions: A comparison of skilled naturalist and lay citizen science recording

**DOI:** 10.1007/s13280-015-0709-x

**Published:** 2015-10-27

**Authors:** René van der Wal, Helen Anderson, Annie Robinson, Nirwan Sharma, Chris Mellish, Stuart Roberts, Ben Darvill, Advaith Siddharthan

**Affiliations:** Aberdeen Centre for Environmental Sustainability, School of Biological Sciences, University of Aberdeen, Aberdeen, AB24 3UU UK; School of Biological Sciences, University of Aberdeen, Aberdeen, AB24 3UU UK; Department of Arctic and Marine Biology, Faculty of Biosciences, Fisheries and Economics, University of Tromsø, 9037 Tromsø, Norway; dot.rural Digital Economy Hub, University of Aberdeen, Aberdeen, AB24 5UA UK; Computing Science, University of Aberdeen, Aberdeen, AB24 3UE UK; Bees, Wasps and Ants Recording Society, 1 Waterloo Road, Salisbury, Wiltshire SP1 2JR UK; Bumblebee Conservation Trust, Beta Centre, Stirling University Innovation Park, Stirling, FK9 4NF UK; British Trust for Ornithology, University of Stirling, Stirling, FK9 4LA UK

**Keywords:** BeeWatch, Biological recording, Bumblebees, Citizen science, National Biodiversity Network, Species distribution

## Abstract

To assess the ability of traditional biological recording schemes and lay citizen science approaches to gather data on species distributions and changes therein, we examined bumblebee records from the UK’s national repository (National Biodiversity Network) and from BeeWatch. The two recording approaches revealed similar relative abundances of bumblebee species but different geographical distributions. For the widespread common carder (*Bombus pascuorum*), traditional recording scheme data were patchy, both spatially and temporally, reflecting active record centre rather than species distribution. Lay citizen science records displayed more extensive geographic coverage, reflecting human population density, thus offering better opportunities to account for recording effort. For the rapidly spreading tree bumblebee (*Bombus hypnorum*), both recording approaches revealed similar distributions due to a dedicated mapping project which overcame the patchy nature of naturalist records. We recommend, where possible, complementing skilled naturalist recording with lay citizen science programmes to obtain a nation-wide capability, and stress the need for timely uploading of data to the national repository.

**Electronic supplementary material:**

The online version of this article (doi:10.1007/s13280-015-0709-x) contains supplementary material, which is available to authorized users.

## Introduction

For centuries field naturalists have been gathering large amounts of biological information, on their own accord and through natural history societies and recording clubs (Burnett et al. 1995; Miller-Rushing et al. 2012). This wealth of records, together with those gathered by professional botanists and zoologists, has shaped our understanding of species distribution and abundance worldwide (Schmeller et al. 2008; Mackechnie et al. 2011). Personal motivations for gathering such data and for making records available to others are numerous but key aspects include the honing of (identification) skills, obtaining social gains from sharing experiences with other recorders, and the enacting of personal relationships with nature and contributing to its protection (Bell et al. 2008; Lawrence and Van Turnhout 2010; Ellis 2011). While the actions of such personal motivations have resulted in the generation of large amounts of biological records, the collation and communication of such information have often been at the local and regional levels, with examples of strong national capacity largely constrained to the most popular of species groups, such as birds and higher plants (e.g., eBird,^1^ Botanical Society of Britain & Ireland^2^).

Growing environmental concern, along with its expression in key international legislative frameworks, such as the 1975 Ramsar Convention, 1983 Bonn Convention and most notably the 1993 Convention on Biological Diversity, drew policy attention to the wealth of biological records that had been gathered for many years. The need to record and monitor species was strongly implicit in these conventions, and fuelled proposals for national capabilities in biological record gathering and reporting around the World (Burnett et al. 1995). Consequently, funding was made available to create and maintain infrastructures to fundamentally change the capacity of countries to bring together biological records and exploit these for local (planning) and national (state of nature) reporting obligations (Lawrence 2010; DEFRA 2014a). However, as was observed for the UK, “Almost the only component of biological recording which has remained relatively constant is the most important source of data, the volunteer specialists and biological societies” (Burnett et al. 1995).

While national data-gathering capacities increased, the number of amateur and professional experts for most species groups declined (Hopkins and Freckleton 2002) and natural history societies saw their membership age (Lawrence 2010). Policy interest in biological records as well as recorders, however, was greater than ever due to heightened concern about biodiversity loss (World Summit on Sustainable Development, Johannesburg 2002). By this time it was recognised at the European policy level that “volunteers both provide support to sampling activities essential for biodiversity monitoring and […] play an important role for the public awareness of biodiversity issues.”^3^ Proliferation of the Internet and associated technologies provided a possible solution to the drop in naturalist numbers at a time when the need for them was greater than ever. The web allowed for the entry of a new contributor: people with an interest in nature but without the high levels of field and identification skills that typified much of the naturalist recording communities (Silvertown 2009). Recording schemes that called on mass participation through so-called ‘citizen science’ initiatives saw a rapid expansion. Such schemes, in which members of the public volunteer species distribution data and thus effectively become ‘human or citizen sensors’ (Catlin-Groves 2012), have demonstrated their value in terms of capturing dynamic biological patterns (Goffredo et al. 2010; Roy et al. 2012a; Pocock and Evans 2014) and are actively promoted (Roy et al. 2012b). Thus, it is evident that as the number of specialist recorders decline, the more generalist volunteer is gradually stepping into their place, although the latter remain dependent on input from a small number of experts for identification or verification of records.

Having arrived at this intersection, we asked the question how the capacities to map species distributions compare between more traditional biological recording and a ‘human sensor’-based lay citizen science approach. We selected bumblebees as a focal species group because their importance to society as pollinators is becoming increasingly recognised (UK NEA 2011; DEFRA 2014b), as is their decline across much of the western world (reviewed in Goulson 2010), thus necessitating good distribution data. To capture naturalist (and potentially also professional) recording activity, we investigated spatial patterns of bumblebee records submitted to the UKs national biodiversity network (NBN), a highly successful repository holding more than 100 million animal and plant species records by the end of 2014. We used the young, but growing, photo submission-based recording programme BeeWatch to obtain citizen science records from largely lay participants.

## Materials and methods

### National Biodiversity Network (NBN)

The NBN was established in 1997 to collate and facilitate the use of biological records at the national level, and to provide information for monitoring reports demanded by the Convention on Biological Diversity (Lawrence and Van Turnhout 2010). Aided by government funds it developed the NBN Gateway; this evolved into a highly comprehensive and sophisticated repository of biological records which are “freely and easily available to everyone.”^4^ The NBN aims to collate data of many different species groups (and habitats) collected by recording schemes and societies across the UK in a standardised electronic format. These data are made available through the NBN Gateway to fulfill a number of aims, which include encouraging public engagement with nature and providing data to assist in land management, conservation and policy-making and inform planning and development schemes.

### BeeWatch

BeeWatch was developed by researchers from the University of Aberdeen (including the authors RvdW, NS, CM and AS of this study) in partnership with the Bumblebee Conservation Trust (BBCT), with its digital portal^5^ launched in August 2011. BeeWatch relies on members of the public to submit photographs of bumblebees, together with location and date of the sighting, via an online interface. Participants are encouraged to identify the bumblebee(s) in the photos they submit with the aid of an online key. Photo submissions are verified by dedicated staff at the BBCT and University of Aberdeen, and automated feedback is provided to the user (Blake et al. 2012). Upon verification, records are stored in a database which is used to generate up-to-date species maps. Through BeeWatch the BBCT aims to contribute to the mapping of bumblebee distributions across the UK to aid conservation, raise societal interest in this species group, and provide tools that allow people to learn to identify bumblebee species. By the end of 2014, over 10 000 photo-records had been submitted to (and verified by) BeeWatch.

### Bumblebee records

**Fig. 1:**
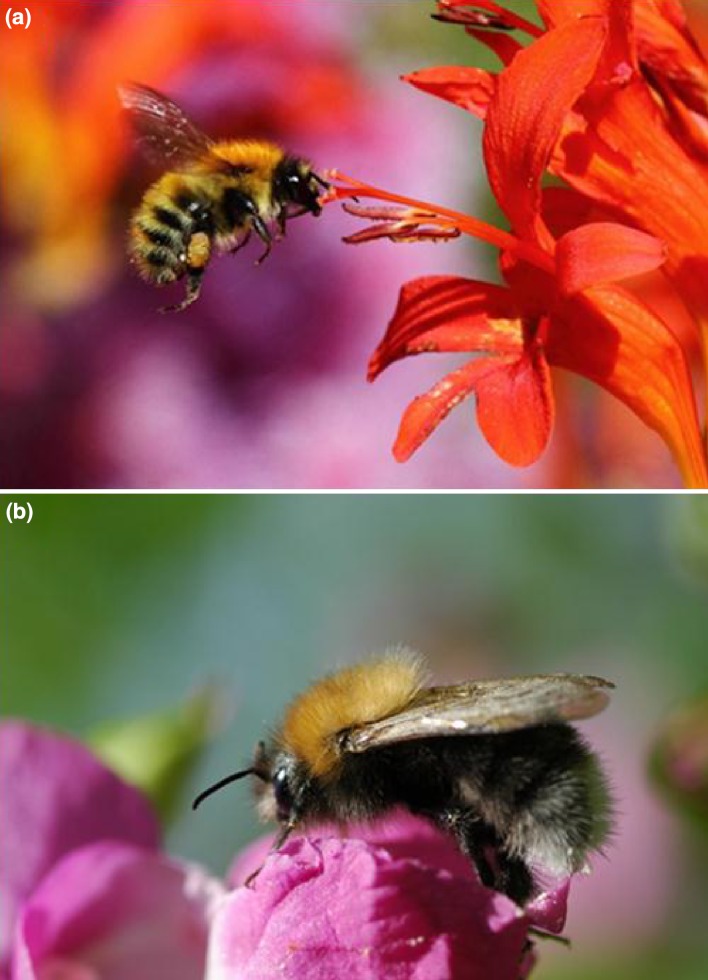
The two focal species of our investigation: **a** the common carder (*Bombus* *pascuorum*), one of the most common species of bumblebee in the UK and relatively easy to identify, although confusion with other carders may occur; and **b** the tree bumblebee (*Bombus hypnorum*), a relative newcomer to the UK, which occurs in gardens and is conspicuous and thus easy to identify. Both images were submitted to the lay citizen science initiative BeeWatch

We obtained all NBN records of bumblebee species across the UK via their online gateway^6^ and contacted the five recording groups for which the Gateway indicated that data was only available on request (resulting in four more data sets). BeeWatch records was obtained from their database (on 26/2/2015). Data from BeeWatch was available at the 100 m scale, while the public access data from NBN was provided at a range of different scales (100 m, 1 km, 2 km, and 10 km), dependent on recording group. For the 4 years where records were available for both programmes (2011–2014), the abundance of each species as a percentage of all bumblebee records held by the NBN or BeeWatch respectively, was calculated (no BeeWatch records had been submitted to the NBN before we downloaded data from their gateway). This allowed us to identify which species (of interest and with sufficient records) to select for subsequent in-depth spatial analysis to determine potential differences between the two recording approaches. As such, we were able to focus our investigation on the two most commonly recorded species (Fig. 1): the widely distributed common carder (*Bombus pascuorum*) (Alford 1980); and the tree bumblebee (*Bombus hypnorum*), a successful newcomer to the UK (first record in 2001—Goulson and Williams 2001). To capture the expansion of the tree bumblebee and annual differences in the distribution of the common carder, we used NBN data for the period 2002–2014 and BeeWatch data for 2011–2014.

To gain further depth, we obtained additional tree bumblebee data for the period 2011–2014 from two sources. First, we were provided access (in December 2014) to all photos of tree bumblebees that were submitted to the Open Air Laboratories (OPAL) as part of their Bugs Count survey or separately.^7^ We verified all those from 2013–2014; 2011–2012 photos had already been verified and corresponding records were provided by OPAL. These data allowed us to compare BeeWatch with another ‘lay citizen science recording’ approach. Second, we obtained the latest tree bumblebee data from the Bees, Wasps and Ants Recording Society (BWARS; on 26/2/2015), which runs a dedicated mapping project for this species.^8^ Our analysis revealed that BWARS was the main provider of tree bumblebee records to the NBN; however, BWARS records of the final year of our investigation (2014) had not been submitted by the time we downloaded the NBN data. Having obtained access to the most up-to-date BWARS data allowed us to compare the distribution of records obtained by two sets of naturalist recorders: those gathered by BWARS and those who submit their records directly to the NBN.

### Spatial data

To aid our spatial analyses of bumblebee records, we used information on Watsonian vice-county boundaries^9^ to map Local Records Centre (LRC) boundaries. We used data from the Association of Local Environmental Records Centres^10^ and the Biological Recording in Scotland scheme^11^ to confirm LRC spatial boundaries in England, Wales and Scotland. Where boundaries for current LRCs differed from the Watsonian vice-county boundaries, these were redrawn in ArcMap (Supplementary material, Fig. S1).

### UK human population data

To determine the potential influence of human population size on the number of bumblebee records, we obtained population counts from the 2011 UK census data.^12^^,^^13^ Since granularity of the census data was at the electoral ward scale (small geographical areas used for local government administration), we joined (in ArcMap) the 2011 census data with electoral ward boundary data^14^ to provide spatial data on human population sizes across the UK at the electoral ward level. Merging all electoral wards which lay within a single LRC region and then spatially joining the bumblebee records from the NBN and BeeWatch to these areas allowed us to obtain the number of bumblebee records, area (in km^2^), and human population size of each LRC region.

### Statistical methods

All spatial analyses were carried out in ArcMap (v10.2.2 ESRI Inc. 1999–2014) and statistical analyses in R (v3.1.1; R Core Team 2014). To simplify (GIS-based) spatial analyses, we only covered records on mainland UK, therefore excluding all offshore islands and Northern Ireland. To be able to compare how close the individual bumblebee sightings were to each other for the different recording approaches (NBN vs. BeeWatch), nearest neighbour distances for each year were calculated in ArcMap. These were subsequently compared, for each year separately, to nearest neighbour distances of a randomly generated distribution of records across mainland UK, where values less than 1 indicate clustering and values equal to 1 indicate a random distribution. To determine if the randomly generated and actual record distributions differed in any one year, we compared their means using z-tests, allowing us to state if the records were clustered or followed a random distribution pattern. The area of mainland UK (in km^2^) covered by bumblebee records in any one year for each recording approach was calculated from the minimum bounding geometry, which enclosed all recorded sightings for that year.

To determine whether the number of bumblebee records per LRC region was related to the size of its human population, we used the glmmADMB package to fit generalised linear mixed models with a negative binomial distribution and log link function; this was done by recording approach (NBN/BeeWatch), and for common carder and tree bumblebee separately. LRC region was modelled as a random effect and year, human population size, and size of LRC region as fixed effects.

To avoid confounding naturalist recording with lags in data provision, we explicitly conducted analyses of NBN data for both 2011–2012 (to represent complete as possible naturalist data) and 2011–2014 (the full period for which data could be compared with BeeWatch). Descriptive NBN data were either given by year (species maps) or time period (2011–2012 and 2013–2014) to facilitate interpretation of both naturalist recording capacity and (observed—see ‘Results’ section) delays in data provision to the NBN.

## Results

### Relative abundance of bumblebee species

**Fig. 2:**
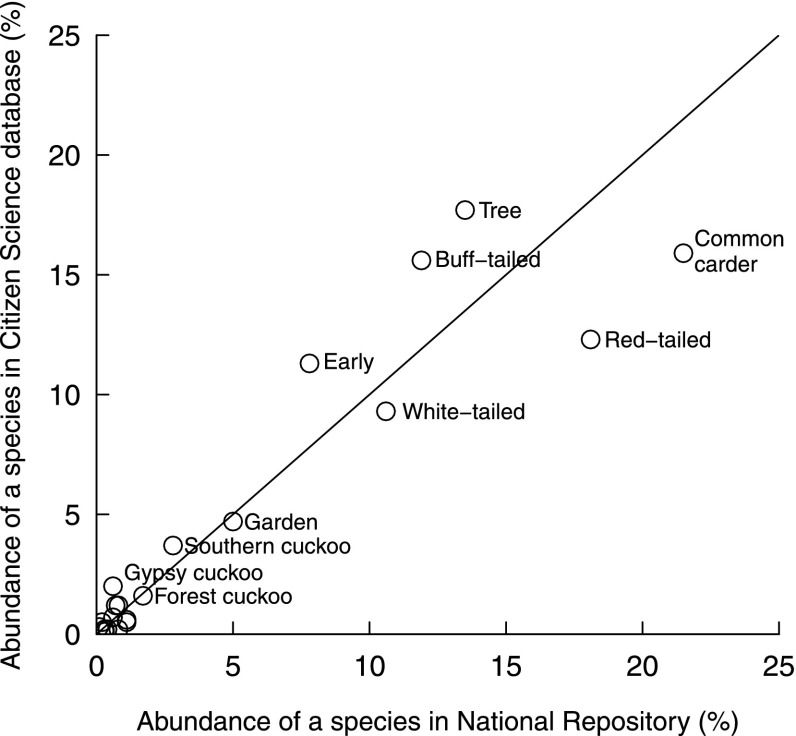
Relative abundance of UK bumblebees based on naturalist records captured by the UK’s national repository (National Biodiversity Network) and records from a lay citizen science initiative (BeeWatch). Data cover the period 2011–2014. The closer a point is to the *diagonal line* (*y* = *x*), the more similar the relative abundance of that species was for both recording approaches

For the 4 years (2011–2014) for which a direct comparison could be made between traditional recording scheme data (on NBN) and citizen science data (on BeeWatch), all 22 bumblebee species were recorded through both approaches and with broadly similar relative species abundances (Fig. 2; *r* = 0.93). The three species with the most records on the NBN were common carder (22 % of all records), red-tailed bumblebee (*B. lapidarius*—18 %) and tree bumblebee (14 %); on BeeWatch these were tree bumblebee (18 %), common carder (16 %) and buff-tailed bumblebee (*B. terrestris*—16 %). Of the rarer species, there was again reasonable agreement about individual species’ relative abundance (Fig. 2); 17 % of NBN records were of species with a relative abundance of 5 % or less, compared to 18 % on BeeWatch. However, some species had greater representation on the NBN (e.g., bilberry bumblebee—*B. monticola*) and others on BeeWatch (e.g., gypsy cuckoo—*B. bohemicus*).

### Spatial distribution of common carders

For the common carder, the NBN held 21 441 mainland UK records between 2002 and 2014 (Table 1a). The annual number of records fluctuated in the period 2002–2012 (between 1077 and 2317) but with evidence for a decline (*z* = −5.80, *p* < 0.001); also the spatial area covered by these records decreased significantly (*z* = −10.4, *p* < 0.001).^15^ Numbers were lower for 2013 and notably 2014 due to delays in data reporting to the NBN.

**Table 1:** Total number of records, cluster analysis results [*z* score and associated significance level (*p* value)], and the total area of mainland UK covered by records of (a) the common carder (*Bombus* *pascuorum*) and (b) the tree bumblebee (*Bombus hypnorum*) from the National Biodiversity Network (NBN) and BeeWatch. The number of records on the NBN which were provided by the Bees, Wasps and Ants Recording Scheme (BWARS) is also given (in brackets). The area covered by bumblebee records was calculated from the minimum bounding geometry that enclosed all records for that year. Data accessed from the NBN Gateway on 25/2/2015 and from BeeWatch on 26/2/2015; NBN records include those obtained directly from four record centres (as only available on request)

Year	National Biodiversity Network (NBN)					BeeWatch				
	No. NBN records (BWARS)	Area covered (km^2^)	Nearest neighbour ratio^a^	*z* score^b^	*p* value	No. records	Area covered (km^2^)	Nearest neighbour ratio^a^	*z* score^b^	*p* value
(a) Common carder										
2002	1992 (1225)	202 883	0.15	−68.1	<0.001	–	–	–	–	–
2003	1982 (1000)	214 166	0.15	−72.1	<0.001	–	–	–	–	–
2004	2317 (1215)	212 015	0.18	−75.6	<0.001	–	–	–	–	–
2005	1668 (803)	193 659	0.19	−63.6	<0.001	–	–	–	–	–
2006	1699 (505)	161 343	0.13	−68.4	<0.001	–	–	–	–	–
2007	2165 (649)	155 997	0.12	−78.0	<0.001	–	–	–	–	–
2008	1835 (583)	159 422	0.13	−71.3	<0.001	–	–	–	–	–
2009	1959 (857)	153 425	0.14	−72.7	<0.001	4	–	–	–	–
2010	1077 (400)	106 822	0.21	−49.9	<0.001	1	–	–	–	–
2011	1695 (521)	153 082	0.17	−65.0	<0.001	29	100 771	0.52	−5.0	<0.001
2012	1569 (175)	136 382	0.20	−61.3	<0.001	318	165 409	0.44	−17.4	<0.001
2013	1130 (24)	126 500	0.20	−51.4	<0.001	465	169 338	0.55	−18.2	<0.001
2014	353 (0)	93 027	0.20	−28.9	<0.001	290	139 489	0.39	−19.8	<0.001
(b) Tree bumblebee										
2002	1 (1)	–	–	–	–	–	–	–	–	–
2003	0 (0)	–	–	–	–	–	–	–	–	–
2004	4 (4)	–	–	–	–	–	–	–	–	–
2005	10 (9)	15 434	−0.66	−2.1	0.04	–	–	–	–	–
2006	26 (25)	9930	0.26	−7.2	<0.001	–	–	–	–	–
2007	129 (122)	41 343	0.17	−18.1	<0.001	–	–	–	–	–
2008	166 (136)	36 213	0.25	−18.6	<0.001	–	–	–	–	–
2009	753 (636)	72 012	0.18	−43.0	<0.001	–	–	–	–	–
2010	1247 (1087)	105 722	0.26	−50.3	<0.001	1	–	–	–	–
2011	743 (392)	97 838	0.31	−36.0	<0.001	22	40 680	0.87	−1.1	0.26
2012	1457 (989)	125 645	0.31	−50.8	<0.001	245	107 075	0.55	−13.4	<0.001
2013	528 (26)	58 230	0.31	−28.8	<0.001	436	118 958	0.64	−14.5	<0.001
2014	327 (0)	32 429	0.29	−24.5	<0.001	525	122 638	0.43	−24.3	<0.001

^a^The nearest neighbour ratio is the observed spatial distribution of records compared to an expected random spatial distribution of records. Values less than 1 indicate clustering and values equal to 1 indicate a random distribution

^b^The test statistics are the results of z-tests comparing the means of a randomly generated distribution of records with the actual distribution of records

**Fig. 3:**
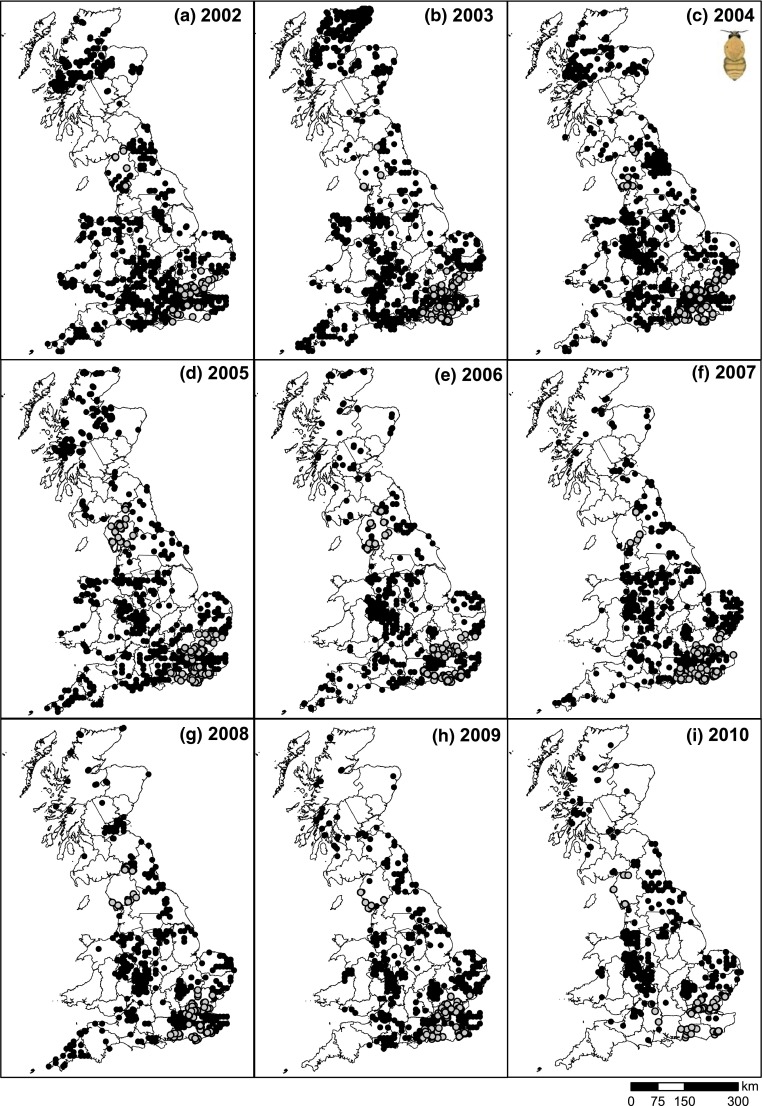
Spatial distributions of common carder (*Bombus pascuorum*) records on mainland UK for 2002–2010 as captured by the UK’s national repository (National Biodiversity Network). Data accessed from the NBN Gateway on 25/2/2015. *Grey circles* indicate records which were requested directly from the provider (as data were only available on request)

**Fig. 4:**
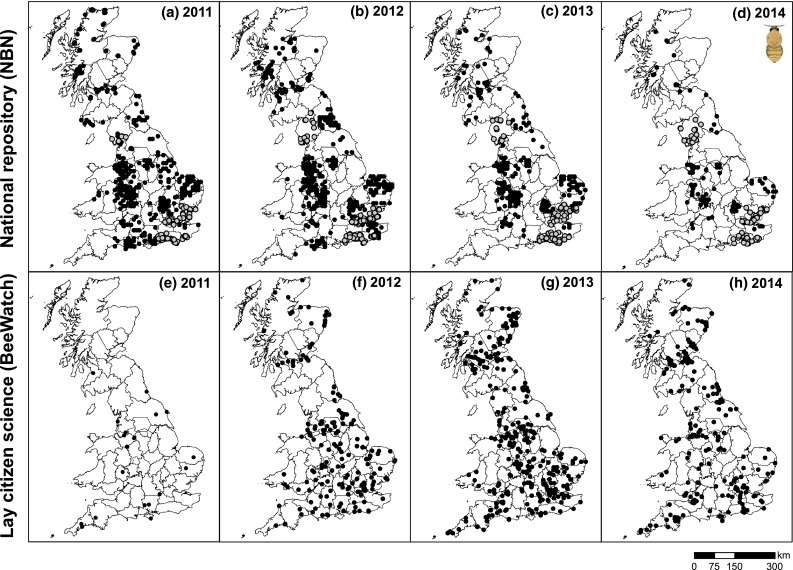
Spatial distributions of common carder (*Bombus pascuorum*) records on mainland UK for 2011–2014 as captured by the UK’s national repository [National Biodiversity Network; (**a**–**d**)] and a lay citizen science initiative [BeeWatch; (**e**–**h**)]. Data accessed from the NBN Gateway on 25/2/2015 and from BeeWatch on 26/2/2015. *Grey circles* indicate records which were requested directly from the provider (as data were only available on request)

When comparing the spatial distribution of common carder records year by year (Fig. 3), it is evident that record provisions are not consistent through time. For example, in 2003, many records were returned from northern Scotland, while in other years numbers of records were very low. Likewise, for NW Wales, records were only forthcoming during 2002–2005, and a large number of records were obtained from Newcastle and surroundings only in 2004. Indeed, when inspecting the provenance of the NBN data, it became clear that few record providers were active in both early and late periods. The relative contribution of the Bees, Wasps & Ants Recording Society (BWARS) dropped from 2224 records in 2002–2003 (56 % of the total number of records) to only 710 (22 %) in 2011–2012 (Table S1), reflecting the gradual decline in the number of records of this species submitted from BWARS to the NBN. The number of records contributed by the second most prolific provider in 2002–2003 (Highland Biological Recording Group) dropped from 330 (8 % of all records on the NBN) to 53 (2 %), while the Bedfordshire- and Luton-based centre stepped up from two records in 2002–2003 to an impressive 553 (16 %) in 2011–2012. The drop in the number of records from notably BWARS, but also several local recording organisations, has meant that for the last 5 years records of this ubiquitous species were rather patchy (Figs. 3, 4), although the pattern for notably 2014 was compounded by delays in naturalist record provision to the NBN.

BeeWatch gathered 1102 mainland UK records of common carder between 2011 and 2014, with the number of sightings increasing from 29 in 2011 to 290 in 2014 (Table 1a). The expansion of BeeWatch records was due to a series of press campaigns which led to an increase in the number of photo submissions by new contributors, as well as reactivating individuals that had previously submitted photos. During 2014, the last year of this investigation, little press activity took place, and the number of common carder records (and the spatial area of mainland UK covered) fell accordingly (Table 1a).

Although the overall number of common carder records on the NBN was higher than on BeeWatch (e.g., almost four times as high in 2012), the latter appeared far more dispersed (Fig. 4) and covering a larger part of mainland UK (Table 1a). Indeed, while records for both NBN and BeeWatch were clustered, the lower nearest neighbour ratios for NBN records (all <0.22) indicated a considerably higher degree of clustering than was the case for BeeWatch records (all >0.38; Table 1a).

### Spatial distribution of tree bumblebees

The NBN held 5391 mainland UK records of tree bumblebees between 2002 and 2014 (Table 1b). The number of records increased sharply from 4 in 2004 to a peak of 1457 in 2012, thereby reflecting the rapid expansion of the species across much of south and middle England. Thereafter, the number of records on the NBN dropped to below 530 per year (Table 1b) due to reduced data reporting rather than a species decline; the spatial area covered by the records of tree bumblebees on the NBN followed a similar pattern.

**Fig. 5:**
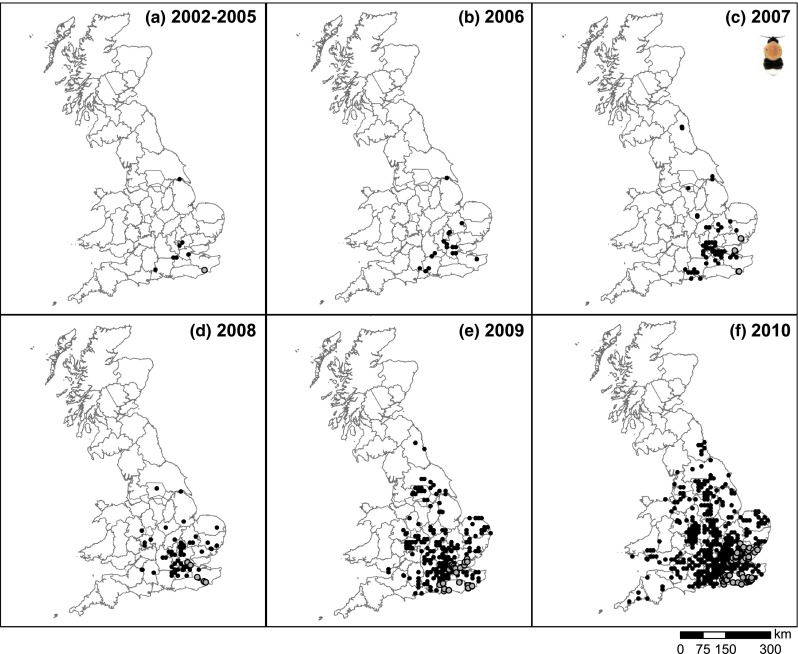
Spatial distributions of tree bumblebee (*Bombus hypnorum*) records on mainland UK for 2002–10 [first 4 years combined (**a**), thereafter by year (**b**–**f**)] as captured by the UK national repository (National Biodiversity Network). Data accessed from the NBN Gateway on 25/2/2015. *Grey circles* indicate records which were requested directly from the provider (as data were only available on request)

**Fig. 6:**
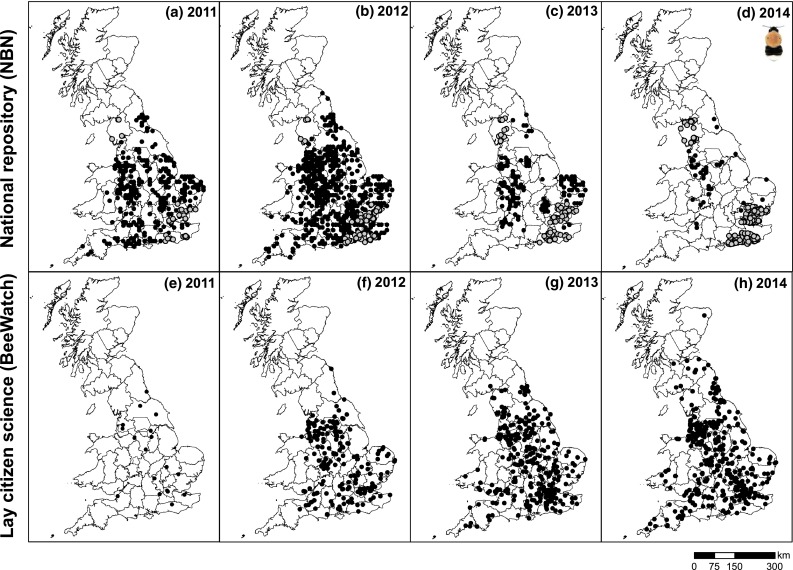
Spatial distributions of tree bumblebee (*Bombus hypnorum*) records on mainland UK for 2011–2014 as captured by the UK national repository [National Biodiversity Network; (**a**–**d**)] and a lay citizen science initiative [BeeWatch; (**e**–**h**)]. Data accessed from the NBN Gateway on 25/2/2015 and from BeeWatch on 26/2/2015. *Grey circles* indicate records which were requested directly from the provider (as data were only available on request)

The spatial pattern of expansion up to and including 2012 appeared to show a progressive invasion from the south-east of England (Figs. 5, 6), with the exception in 2011 due to data flow issues between BWARS and the NBN. Indeed, this society contributed the vast majority of tree bumblebee records found in the NBN database (e.g., 87 % for 2002–2010) due to its dedicated mapping project. BWARS was thus acting as a gatekeeper, i.e., gathering records from others, and verifying those, before passing them on to the NBN; and by doing so capturing the expansion of this new species across the UK. However, there were also providers who submitted their records directly to the NBN throughout the study period (e.g., Bedfordshire, Norfolk, and Essex based recording centres; Table S2). The records of several of these providers increased by an order of magnitude between 2002 and 2012, reflecting the increase in geographic spread and local abundance of this species (which in turn reduced the contribution by BWARS to 63 % for 2011–2012). The spatial patterns for 2013–2014 were fragmented, signaling delays in record provision rather than a change in fortune of the tree bumblebee (see below).

BeeWatch gathered a total of 1228 mainland UK records of tree bumblebees between 2011 and 2014 (Table 1b). The number of records was low in the first year of the scheme (22 in 2011—because BeeWatch only went live in August, thus just capturing the end of the main bumblebee season), but thereafter increased sharply to more than 500 records in 2014 (Table 1b); the spatial area covered by tree bumblebee records likewise expanded over the 4 years (Table 1b). Due to the short period of time in which BeeWatch has been running, these results likely reflect increased participation in the BeeWatch initiative as much as an actual expansion of the species across the UK.

The number of tree bumblebee records on the NBN was far greater than on BeeWatch. Nevertheless, the area over which the records were gathered was generally largest for BeeWatch (Table 1b). Also while both data sources exhibited significant clustering (all *p* < 0.001), the NBN data showed a higher degree of clustering (nearest neighbour ratios all <0.32, Table 1b) than the more dispersed BeeWatch sightings (nearest neighbour ratios >0.42).

### Relationships between bumblebee records and human population size

**Fig. 7:**
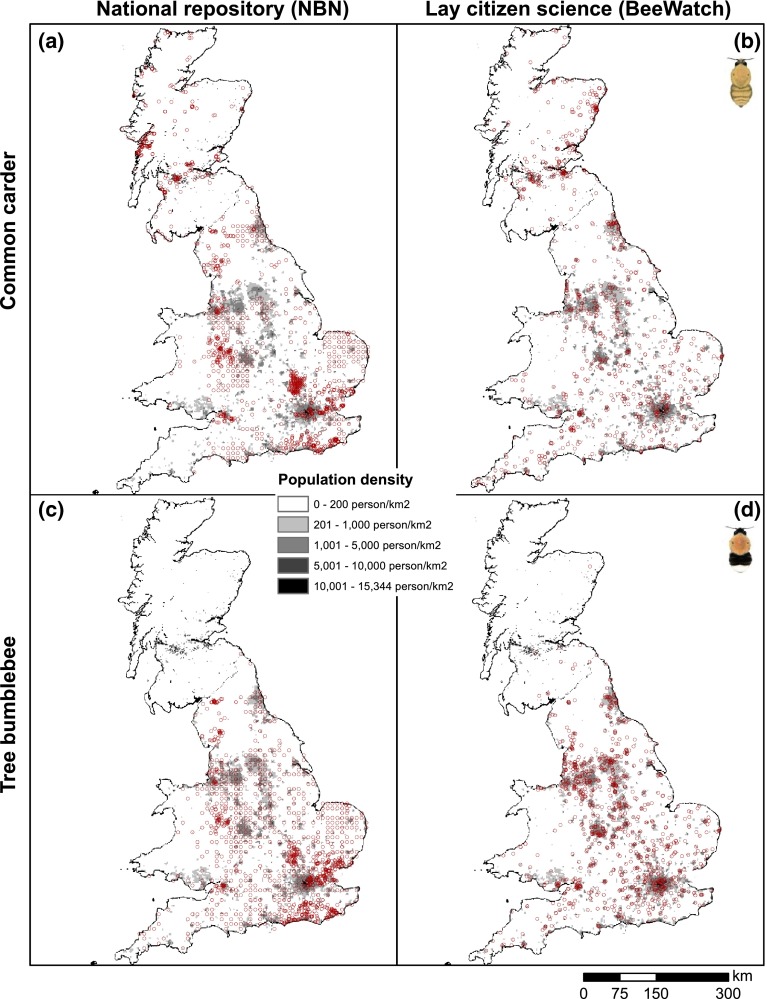
Human population densities (in *grey*) and the spatial distributions of records (in *red*) of common carder [*Bombus pascuorum*; (**a**, **b**)] and tree bumblebees [*Bombus hypnorum*; (**c**, **d**)] from 2011–2014 as captured by the UK’s national repository (National Biodiversity Network) and a lay citizen science platform (BeeWatch). Human population information was obtained from the 2011 UK censuses. Data accessed from the NBN Gateway on 25/2/2015 and from BeeWatch on 26/2/2015

When overlaying the records of either common carder or tree bumblebee on human population density, it was apparent that BeeWatch records came from parts of the country where many more people live (and thereby potential scheme participants), while for NBN this was less evident (Fig. 7). In fact, there were remarkable mismatches between NBN record prevalence and human population density. For example, most Scottish NBN records of common carder came from across the Highlands—an area of distinctly low human population density. By contrast, BeeWatch records from the Scottish Highlands were scarce, with most Scottish records coming from populated lowland areas (including the densely populated areas around Glasgow, Edinburgh and Aberdeen). Formal analysis revealed that for NBN data there was no significant relationship between the number of common carder records from a Local Recording Centre (LRC) and the size of its recording area or human population size (all *p* > 0.2—Table 2), while for BeeWatch records both predictors were highly significant (*p* < 0.003), with more common carder records coming from recording regions which were larger in size and harboured more people. For tree bumblebee, there was no such stark difference between NBN and BeeWatch, particularly when concentrating on the years 2011–2012 for the NBN only (to avoid confounding naturalist recording with lags in data provision), with highly significant effects of human population size (*p* < 0.01) for both recording approaches. Thus, BeeWatch records and NBN tree bumblebee records reflected the abundance of humans living in an area, and thereby the number of potential record submitters, while NBN common carder records occurred when and where a record centre or group was present or active.

**Table 2:** Summary of the statistical relationships between recording area/population size and the number of records held by the UK national repository (NBN) and BeeWatch for (a) the common carder (*Bombus* *pascuorum*) and (b) the tree bumblebee (*Bombus hypnorum*) from 2011–2014 (2011–2012 in brackets). Size of the LRC region refers to the area of land covered by a particular Local Recording Centre region as derived from Watsonian vice-county boundaries (see Fig. S1 for LRC locations); human population size refers to the number of people recorded as living within a particular LRC region on the basis of census data

	NBN	BeeWatch
(a) Common carder		
Size of LRC region (km^2^)	*z* = 0.92, *p* = 0.36 (*z* = 1.07, *p* = 0.28)	*z* = 4.33, *p* < 0.001
Human population size	*z* = 0.91, *p* = 0.36 (*z* = 0.91, *p* = 0.36)	*z* = 3.06, *p* = 0.002
(b) Tree bumblebee		
Size of LRC region (km^2^)	*z* = −1.71, *p* = 0.09 (*z* = −1.62, *p* = 0.12)	*z* = −0.72, *p* = 0.47
Human population size	*z* = 2.42, *p* = 0.02 (*z* = 2.64, *p* = 0.008)	*z* = 4.29, *p* < 0.001

### Comparing various skilled and lay citizen science approaches

**Fig. 8:**
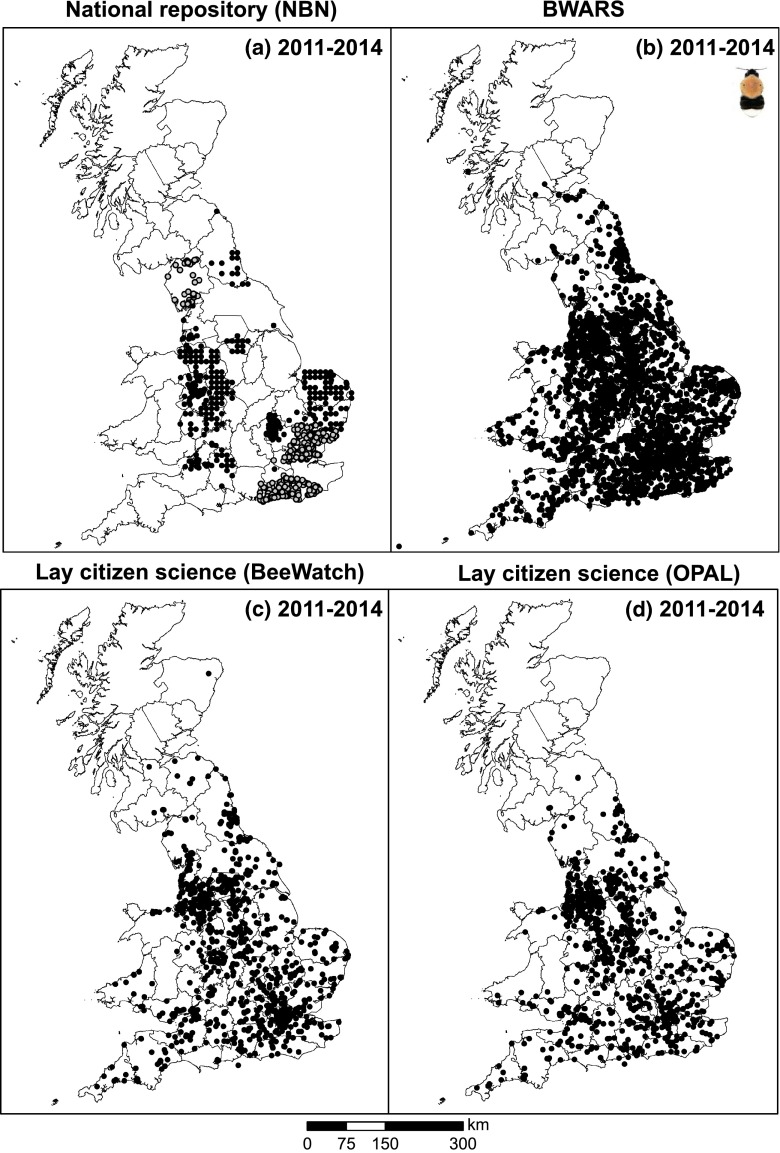
Spatial distributions of tree bumblebee (*Bombus hypnorum*) records on mainland UK for 2011–2014 as captured by **a** the UK’s national repository (National Biodiversity Network), **b** BWARS (Bees, Wasps and Ants Recording Society), and two lay citizen science initiatives (**c** BeeWatch and **d** OPAL). Data accessed from the NBN Gateway on 25/2/2015 (and records from BWARS removed), from BWARS and BeeWatch on 26/2/2015 and from OPAL on 16/12/2014. OPAL data were based on photos of tree bumblebees that were submitted to the Open Air Laboratories as part of their Bugs Count survey, or separately (http://www.opalexplorenature.org/SpeciesQuestBugs). *Grey circles* in **a** indicate records which were requested directly from the provider (as data were only available on request)

The reduced number of 2011 records from BWARS already showed how critically dependent the NBN is on this organisation to capture the spread of tree bumblebee. Indeed, when inspecting those NBN records not provided by BWARS (for the focal period 2011–2014) a very patchy distribution resulted (Fig. 8a), which resemble NBN common carder records (Fig. 4), and sharply contrasts the extensive web of records gathered by BWARS (Fig. 8b; capturing all their records for the period 2011–2014 as directly obtained from the society rather than through the NBN). This contrast was heightened by BWARS naturalists gathering about twice as many records in 2014 (1928) than in the three preceding years (916–1096; Table S3). In fact, the area across which BWARS managed to gather data exceeded that of both BeeWatch and OPAL (Fig. 8c, d; Table 3). Those two lay citizen science initiatives, in turn, revealed a remarkably similar and widespread occurrence of this species across England, despite having very different modus operandi. Comparing all four forms of (naturalist and lay) recording, it is clear that the NBN data without input from BWARS is the ‘odd one out’ (Fig. 8), but also that records from both lay citizen science approaches were less clustered than naturalist records (Table 3).

**Table 3:** Total number of records, cluster analysis results (*z* score and associated significance level (*p* value)), and the total area of mainland UK covered by records of the tree bumblebee (*Bombus hypnorum*) for the four sources of data used to compose Fig. 8

Tree bumblebee (2011–2014)	No. records	Area covered (km^2^)	Nearest neighbour ratio	*z* score	*p* value
NBN (without BWARS)	1648	66 059	0.23	−59.0	<0.001
BWARS	6638	167 111	0.27	−114.0	<0.001
OPAL	956	124 709	0.51	−28.8	<0.001
BeeWatch	1228	145 074	0.48	−34.9	<0.001

## Discussion

Like many European countries, the UK has an extensive network of skilled naturalists who gather an impressive volume of biological records. Many of these records are held by the UK’s national repository, the National Biodiversity Network (NBN), which, after some initial struggles (see Lawrence 2010), has become highly successful. Our investigation of bumblebee records from the NBN and the lay citizen science initiative BeeWatch revealed several limitations with both systems. Here we discuss the most salient aspects of the two underlying approaches to biological recording and evaluate their complementarity. In doing so, we suggest how lay citizen science initiatives may strengthen naturalist recording and thereby improve the mapping of species distributions over time.

### Distribution of observers rather than distribution of species

Our investigation revealed that, at least in England, a sufficiently wide network of naturalists could be relied on to submit records of a focal species to a dedicated species mapping project, thereby capturing the spread of a relative newcomer (tree bumblebee) for well over a decade. Likewise, for the UK’s most common species (common carder), good regional cover was achieved in a few areas but often for only short periods of time. In the absence of incentives, such as regional atlas production or targeted species programmes (see also Tulloch et al. 2013), the naturalist recording network failed to record widely and consistently and was unable to track the distribution of a ubiquitous species, a fate that is likely shared with many other common species and species groups that do not gain widespread popularity (Hopkins and Freckleton 2002).

The most striking difference between the two modes of biological recording, exemplified by records of the common carder, was that the national repository revealed where active record centers were, whereas the lay citizen science initiative reflected where most people lived. Such spatial and temporal bias in species occurrence data tends to be the rule rather than the exception (Moerman and Estabrook 2006; Boakes et al. 2010; Isaac et al. 2014). However, the two recording approaches may complement each other precisely *because* of their differences in geographical bias (Tulloch et al. 2013). For example, our data show that in some sparsely populated areas (such as the Scottish Highlands) the lay citizen science initiative failed to obtain records, while naturalists did (partially due to concerted effort to create the Highland bumblebee atlas; Macdonald and Nisbet 2006). Conversely, few naturalists recorded in inner cities, while lay effort was readily mobilised there. However, in other areas (such as large parts of Wales) neither approach seemed effective; publicising such a knowledge gap may simultaneously encourage dormant naturalists and interested members of the public to contribute to biological recording, thus leading to better geographic coverage.

We found a remarkable similarity in record distribution between two very different lay citizen science initiatives (see Fig. 8), which in turn resembled rather well the spread of records gathered by the network of naturalists contributing to a dedicated mapping scheme. This suggests reasonable level of robustness of lay data gathering—providing adequate verification procedures are in place—and adds value to those studies that reveal strong spatial agreement between expert and citizen science recording in a systematically derived setting (e.g., Paul et al. 2014).

### Transient recorder effort

Our investigation also showed that both naturalist and lay citizen science recording were transient in nature. A great amount of publicity is required to start a lay citizen science initiative (for guidance see, e.g., eBird; Roy et al. 2012b), and thereafter many struggle with maintaining interest over prolonged periods of time. Since BeeWatch is a relatively new initiative it would have to run for much longer to genuinely track changes in species distribution over time. This would require a combination of continued advertising through mass media (to generate new submitters and rekindle interest among previous contributors) and having in place a series of instruments (e.g., motivational training, fostering of social interactions over identifications), which would allow for the forming of a community of BeeWatch users. However, if accomplished, this could arguably be viewed as a (virtual) modern day equivalent of a natural history society or recording group (and partially address the issue of shrinking and aging traditional recorder networks—Hopkins and Freckleton 2002; Lawrence 2010).

The series of common carder maps laid bare the absence of a consistent national capacity and reflected where specific record centres, groups and individuals were active and when, rather than where this ubiquitous species could be found. Only by aggregating data over many years, as is commonly done to communicate a species’ distribution, is it possible to generate a fairly comprehensive national picture, be it at relatively low resolution due to data scarcity. Despite considerable progress in modelling noisy species record data (e.g., Barbet-Massin et al. 2012; Beck et al. 2014; Isaac et al. 2014; Kelling et al. 2015), capturing spatial distribution trends on the basis of naturalist records over shorter (<10 year) time scales remains a challenge. Hence, addressing even rather basic questions such as “are distributions of our most common species of bumblebee shrinking” becomes very difficult. This became apparent for the common carder where we observed a rather striking decline in the number of records offered to the NBN since the first year of our investigation (2002). While the species may well be less frequent than it used to be (Lye et al. 2011), we doubt that this is the reason behind the reduction in the number of records over the years (notably from BWARS). Instead, we suggest that recording effort of rarer or range-expanding species may have been at the expense of species, such as the common carder, which are thought to be abundant and occur over a wide geographical range (Alford 1980).

### Recording species of interest

Naturalists often show a bias towards recording rare or unusual species (Greenwood 2007), while lay citizen science initiatives are often portrayed as best suited for recording easily identifiable common species (Roy et al. 2012b). However, despite a recent evaluation of citizen science data on pollinator communities showing that many species were indeed missed in the field by lay recorders (Kremen et al. 2011), our investigation indicated that differences in the relative abundance of bumblebees calculated from NBN and BeeWatch records were remarkably slim. Moreover, some of the more apparent differences were likely due to the sampling of different habitats: there was a greater prevalence of the urban dwelling tree bumblebee (Crowther et al. 2014) in BeeWatch in line with greater recorder effort in urban areas; and fewer bilberry bumblebee (*B. monticola*) records were on BeeWatch (compared to NBN) pointing at low lay citizen science recorder effort in the mountains. Because BeeWatch is a photo-based citizen science initiative, limited identification skills are not necessarily an obstacle (as provided by experts). Hence, the absence of large differences in the relative abundance of species between the two approaches (Fig. 2) suggests that lower levels of field skills (BeeWatch) do not necessarily lead to a fundamentally different level of decision-making when it comes to what to photograph or record.

### Timely portrayal of biological records

NBN data for the final 2 years of our study (2013–2014) were highly incomplete, thus revealing that the process of record provision to the national repository was rather slow. A series of issues were identified, with data handling by organisations, data flows between them and verifying records being the most important. Verification of the many records BWARS handles was a key factor behind timely delivery of records to the NBN. Yet BWARS fulfills the important role, on a voluntary basis, of gate keeper; it has some of the best experts of this (and other) species groups in the country, and thus brings quality assurance to bumblebee recording. Most other data providers to the NBN (Tables S1, S2) are funded by local governments and are notoriously short-staffed. Hence, to upload all records gathered from across the network of naturalists in their area during a year is a mammoth task, thus inevitably leading to a delay in making the records available to the NBN; added to this is the inevitable time needed for NBN staff to handle the large volumes of data coming in. The consequence of these series of delays is, however, that species distributions are out-of-date by one or more years. Finally, several providers did not allow their data to be visible on, or downloadable through, the NBN Gateway. While replies to requests for this data were swift and successful in four out of five cases, thus allowing us to have the most up-to-date information, it is clear that none of this recording effort is visible to the nation.

### The future of biological recording

The NBN serves naturalist interests well by bringing data together and combining a relatively large number of years, thus leading to informative species distribution maps. Yet, its limited national capacity and relatively slow procedures for data portrayal arguably do not allow ‘a finger on the pulse’ of UK biodiversity and thus may frustrate policy development and conservation action (Ellis and Waterton 2005). Lack of timely reporting of data prevents or delays the identification of rapid changes in species distributions. While distribution maps of the past may almost have been viewed as species attributes, pressures on once common species may lead them to rapidly gain rarity status (e.g., Lindenmayer et al. 2011). We argue that, given the range of pressures on wildlife in general, and concerns about the fate of our pollinators in particular (Goulson 2010; DEFRA 2014b), a national capacity that allows for rapid disclosure of species distribution data is urgently needed. These, in turn, need to be complemented by other structured and repeat surveys (e.g., the BTO’s Breeding Bird Survey^16^; BBCT’s BeeWalk^17^) to provide robust evidence of changes in abundance.

While simple combining of such data will lead to more comprehensive distribution maps, the respective sampling strategies are widely different. Hence, for temporal investigation on, for example, the NBN, the nature of programmes and their biases—where known—would need to be communicated in order for distribution maps to be interpretable. Yet robust integration would require formal modeling approaches that account for differences in sampling strategy. Embedding such approaches within the NBN may lead to lags far in excess of the current delays in data provision to the national repository and runs the risk of making the Gateway’s interface too complicated for general use.^18^

The quality of the relationships between recorders, recording schemes and societies, and the national repository determines to a large extent the biological recording capacity of a nation (Bell et al. 2008; Lawrence 2010), and further study of these relationships is overdue. Governmentally supported national repositories like the NBN are well placed to champion data, given their shear breadth and volume of biological records they hold. However, for the true biological recording capacity of a nation to be reached, capturing distributional change in both common and rarer species, a further meshing of more traditional data-gathering practices with citizen science initiatives needs to take place. This puts pressure on the naturalist recording infrastructure to ensure timely portrayal of its valuable data, and on lay citizen science initiatives to not only make their data readily available to its users, but also to the national repository so that the strengths of both approaches are combined. Eliminating the problem of incomplete and slow flow of biological records would allow species distributions and changes therein to become contemporary knowledge to the benefit of all.

## Electronic supplementary material

Supplementary material 1 (PDF 235 kb)

## Footnotes

1 www.ebird.org.

2 http://www.bsbi.org.uk/.

3 http://www.epbrs.org/PDF/EPBRS-DK2002-Monitoring-_Final_.pdf.

4 http://data.nbn.org.uk/.

5 www.abdn.ac.uk/research/beewatch/.

6 https://data.nbn.org.uk/ [downloaded on 25/2/2015].

7 http://www.opalexplorenature.org/SpeciesQuestBugs.

8 http://www.bwars.com/index.php?q=content/bombus-hypnorum-mapping-project.

9 http://www.nbn.org.uk/SpecialPages/WVCB-download.aspx.

10 http://www.alerc.org.uk/find-an-lerc-map.html.

11 http://www.brisc.org.uk/Sources.php.

12 http://www.ons.gov.uk/ons/guide-method/census/index.html.

13 http://www.scotlandscensus.gov.uk/ods-web/data-warehouse.html.

14 https://geoportal.statistics.gov.uk/geoportal/catalog/content/filelist.page; http://www.lgbc-scotland.gov.uk/maps/datafiles/index_1995_on.asp.

15 We did not use 2013–2014 for this comparison because numbers for that period were low due to delays in record provision.

16 http://www.bto.org/volunteer-surveys/bbs.

17 http://bumblebeeconservation.org/get-involved/surveys/beewalk/.

18 e.g., Atlas of Living Australia, http://www.ala.org.au/.
