# Supplementary material for: Mapping species distributions: A comparison of skilled naturalist and lay citizen science recording
Source: Ambio. 2015 Oct 27;44(Suppl 4):584–600. doi: 10.1007/s13280-015-0709-x (PMC4623864; doi:10.1007/s13280-015-0709-x)
Supplement: Supplementary file 1 — Supplementary material 1 (PDF 235 kb) [file 13280_2015_709_MOESM1_ESM.pdf]

Ambio

Electronic supplementary material

Title: **Mapping species distributions: A comparison of skilled naturalist and lay citizen science recording**

Authors: René van der Wal, Helen Anderson, Annie Robinson, Nirwan Sharma, Chris Mellish, Stuart Roberts, Ben Darvill and Advaith Siddharthan

Table S1 Numbers of common carder (*Bombus pascuorum*) records provided to the UK national repository (National Biodiversity Network) by the respective recording schemes and societies for three time periods. Comparing the years 2002/03 and 2011/12 shows changes in the volume of 'naturalist records' within and between organisations, whilst comparing 2011/12 and 2013/14 highlights the issue of timeliness of data provision. Data were downloaded from the NBN Gateway on 25/2/2015. *Providers from which data were requested directly (as not downloadable from the gateway) and thus more likely to be up-to-date

Organisation	2002/03	2011/12	2013/14	
Bees, Wasps and Ants Recording Society	2224	710	24	
Highland Biological Recording Group	330	53	26	
Sussex Biodiversity Records Centre	191	274	118	*
Natural Resources Wales	140	0	0	
Cheshire RECORD	120	249	250	
Suffolk Biological Records Centre	120	31	24	
Rotherham Biological Records Centre	118	10	13	
Essex Field Club	117	90	90	*
NE Scotland Biological Records Centre	93	11	0	
Worcestershire Biological Records Centre	86	136	113	
Environmental Records Information Centre North East	85	188	49	
National Trust	74	39	7	
Greenspace Information for Greater London	56	28	81	*
Bristol Regional Environmental Records Centre	51	54	11	
Birmingham EcoRecord	22	0	0	
West Wales Biodiversity Information Centre	16	0	0	
Shropshire Ecological Data Network	15	93	26	
Norfolk Biodiversity Information Service	14	179	30	
South East Wales Biodiversity Records Centre	14	0	0	
Gloucestershire Centre for Environmental Records	12	0	2	
Surrey Biodiversity Information Centre	12	0	0	
Cumbria Biodiversity Data Centre	11	29	46	*
Scottish Wildlife Trust	10	38	6	
Natural England	7	0	0	
Merseyside Biobank	6	81	88	
Thames Valley Environmental Records Centre	6	4	0	
Sheffield Biological Records Centre	3	38	21	
Staffordshire Ecological Record	3	36	4	
Wiltshire and Swindon Biological Records Centre	3	19	8	
Bedfordshire and Luton Biodiversity Recording and Mon. Centre	2	533	285	
Devon Biodiversity Records Centre	2	0	0	
Herefordshire Biological Records Centre	2	26	7	
Lancashire Environmental Record Network	2	45	67	
The Wildlife Information Centre	2	4	0	
Derbyshire Wildlife Trust	1	0	0	
National Trust for Scotland	1	3	13	
Royal Horticultural Society	1	0	0	
Tullie House Museum	1	0	0	
Yorkshire Wildlife Trust	1	5	3	
Buglife - The Invertebrate Conservation Trust	0	42	53	
Cambridgeshire & Peterborough Environmental Records Centre	0	2	0	
Fife Nature Records Centre	0	0	1	
Herts Environmental Records Centre	0	7	11	
Lorn Natural History Group	0	59	3	
North & East Yorkshire Ecological Data Centre	0	0	3	
Open Mosiac Habitat Survey Group	0	148	0	
**TOTAL**	**3974**	**3264**	**1483**	

Table S2 Numbers of tree bumblebee (*Bombus hypnorum*) records provided to the UK national repository (National Biodiversity Network) by the respective recording schemes and societies for three time periods. Comparing the first nine years of NBN records of this species in the UK (2002/10) with 2011/12 shows changes in the volume of 'naturalist records' within and between organisations, whilst comparing 2011/12 and 2013/14 highlights the issue of timeliness of data provision. Data were downloaded from the NBN Gateway on 25/2/2015. *Providers from which data were requested directly (as not downloadable from the gateway) and thus more likely to be up-to-date

**Organisation**	**2002/10**	**2011/12**	**2013/14**	
Bees, Wasps and Ants Recording Society	2021	1381	26	
Sussex Biodiversity Records Centre	82	59	138	*
Worcestershire Biological Records Centre	48	54	41	
Bedfordshire and Luton Biodiversity Recording and Mon. Centre	38	94	60	
Suffolk Biological Records Centre	38	32	14	
Essex Field Club	34	54	139	*
Greenspace Information for Greater London	32	9	65	*
Norfolk Biodiversity Information Service	13	100	31	
Wiltshire and Swindon Biological Records Centre	6	15	3	
Staffordshire Ecological Record	5	52	44	
Bristol Regional Environmental Records Centre	4	35	13	
Royal Horticultural Society	4	1	0	
Shropshire Ecological Data Network	4	47	11	
Rotherham Biological Records Centre	3	34	77	
Sheffield Biological Records Centre	2	19	9	
Highland Biological Recording Group	1	0	0	
Leicestershire and Rutland Environmental Records Centre	1	0	0	
Birmingham EcoRecord	0	0	1	
Cambridgeshire & Peterborough Environmental Records Centre	0	2	0	
Cheshire RECORD	0	96	33	
Cumbria Biodiversity Data Centre	0	13	48	*
Environmental Records Information Centre North East	0	14	20	
Gloucestershire Centre for Environmental Records	0	2	2	
Herefordshire Biological Records Centre	0	7	3	
Herts Environmental Records Centre	0	0	2	
Lancashire Environmental Record Network	0	5	40	
Merseyside Biobank	0	56	21	
National Trust	0	11	12	
North & East Yorkshire Ecological Data Centre	0	0	1	
Open Mosiac Habitat Survey Group	0	8	0	
Yorkshire Wildlife Trust	0	0	1	
**TOTAL**	**2336**	**2200**	**855**	

Table S3 Numbers of tree bumblebee records held by the Bees, Wasps and Ants Recording Society (BWARS) obtained directly from them on 25/2/2015. The table unfolds some of the complexities of biological recording and record flows. For example, tree bumblebee records on BeeWatch were passed on to BWARS only in January 2015 (to contribute to their dedicated mapping project) and thus will likely be offered (by BWARS) to the NBN in February 2016. BWARS held only two years of OPAL data because the other two years were processed by one of the authors (AR) for this paper and were offered to the society only in June 2015. Whilst more than 70% of BWARS records (Total – BeeWatch records) had materialised through 'naturalist recording', lay records had also been provided to them; this included both miscellaneous records and all submissions through iSpot and iRecord. With BeeWatch and OPAL now also providing their tree bumblebee records to BWARS –because of its species mapping project and central role as verifier – the character of the data the Society offers to the NBN is gradually changing from merely naturalist-based to mixed naturalist and lay contributions. *Records excluded for making Fig. 8b – see methods for details

**Data type and source**	**2011**	**2012**	**2013**	**2014**	TOTAL
Naturalist recording					
Single species survey	13	512	618	1281	*2424*
Field records	903	415	224	197	*1739*
BBCT BeeWalk scheme	0	0	254	450	*704*
Subtotal	*916*	*927*	*1096*	*1928*	*4867*
Lay recording					
BeeWatch*	22	242	426	502	*1192*
OPAL*	61	105	0	0	*166*
Miscellaneous	716	601	3	24	*1344*
iSpot/iRecord	31	29	106	261	*427*
Subtotal	830	977	535	787	*3129*

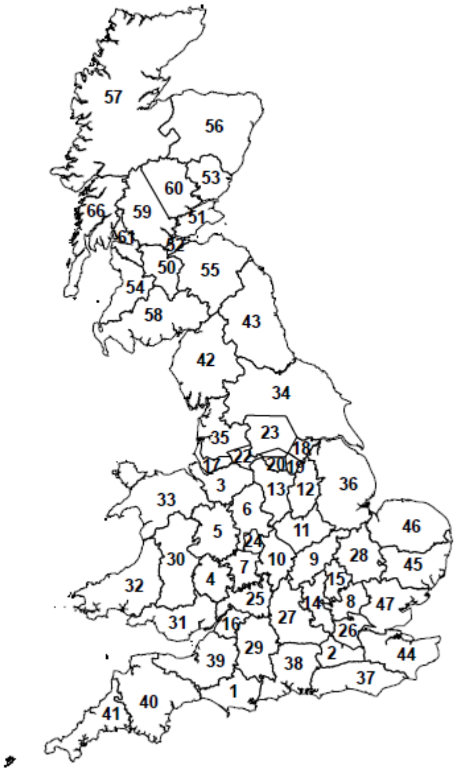

Id	LBRC	Id	LBRC
1	Dorset ERC	31	South East Wales BRC
2	Surrey BIC	32	West Wales BIC
3	Cheshire RECORD	33	North Wales EIS
4	Herefordshire BRC	34	North & East Yorkshire EDC
5	Shorpshire EDN	35	Lancashire ERN
6	Staffordshire ER	36	Lincolnshire ERC
7	Worcestershire BRC	37	Sussex BRC
8	Hertfordshire BRC	38	Hampshire BIC
9	Nothamptonshire BRC	39	Somerset ERC
10	Warwickshire BRC	40	Devon BRC
11	Leicestershire & Rutland ERC	41	ERC Cornwall
12	Nottinghamshire BGRC	42	Cumbria BDC
13	Derbyshire BRC	43	ERIC North East
14	Buckinghamshire & Milton Keynes ERC	44	Kent & Medway BRC
15	Bedfordshire & Luton BRMC	45	Suffolk BRC
16	Bristol RERC	46	Norfolk BIS
17	Merseyside Biobank	47	Essex
18	Doncaster BRC	50	N & S Lanarkshire BRCP
19	Rotherham BRC	51	Fife NRC
20	Sheffield BRC	52	Falkirk
21	Barnsley BRC	53	Naturebase Dundee
22	Greater Manchester LRC	54	Ayrshire BRCP
23	West Yorkshire Ecology	55	WIC Lothian & Borders
24	Birmingham EcoRecord	56	NE Scotland BRC
25	Gloucestershire CER	57	Highland BRG
26	GI Greater London	58	Dumfries Galloway ERC
27	Thames Valley ERC	59	Central Area BRC
28	Cambridgeshire & Peterborough ERC	60	Perth BRC
29	Wiltshire & Swindon BRC	61	Renfrewshire Inverclyde BRC
30	Powys & Brecon Beacons BIS	66	Glasgow Museum BRC

Fig. S1 Map of mainland UK showing the geographic areas covered by individual Local Records Centres (LRCs). Each mainland UK LRC is indicated by a number on the map and identified in the list. Missing numbers (48, 49, 62-65) refer to LRCs located on offshore UK islands, which were not included in this study (and records from those places were thus not included in any of the figures in this paper). Most of the current LRCs follow Watsonian vice-county boundaries, a mapping system devised in the 19th century to record plant distributions throughout the UK (Watson 1852)

References

Watson, H.C. 1852. *Cybele Britannica; or, British plants and their geographical relations*, 3rd edn. London: Longman.
